# Metabolomic and BH3 profiling of esophageal cancers: novel assessment methods for precision therapy

**DOI:** 10.1186/s12876-018-0823-x

**Published:** 2018-06-22

**Authors:** R. Taylor Ripley, Deborah R. Surman, Laurence P. Diggs, Jane B. Trepel, Min-Jung Lee, Jeremy Ryan, Jeremy L. Davis, Seth M. Steinberg, Jonathan M. Hernandez, Choung Hoang, Cara M. Kenney, Colleen D. Bond, Tricia F. Kunst, Anthony Letai, David S. Schrump

**Affiliations:** 1 Thoracic and GI Oncology Branch, Center for Cancer Research, National Cancer Institute, Building 10; 4-3952, 10 Center Drive, MSC 1201, Bethesda, MD 20892-1201 USA; 2 Dana-Farber Cancer Institute, Harvard Medical School, Boston, MA USA; 3 Biostatistics and Data Management Section, Center for Cancer Research, National Cancer Institute, Bethesda, MD USA; 4 Developmental Therapeutics Branch, Center for Cancer Research, National Cancer Institute, Bethesda, MD USA

**Keywords:** Esophageal adenocarcinoma, Esophageal squamous cell carcinoma, Pathologic complete response, BH3 profiling, Metabolomics

## Abstract

**Background:**

Esophageal cancers accounted for nearly 16,000 deaths in 2016. The number of patients with esophageal cancers increases every year. Neoadjuvant chemoradiotherapy (nCRT) prior to esophagectomy is a standard treatment for esophageal cancers. The patients who have no residual tumor (pathological complete response (pCR)) at surgery are the most likely to experience long term survival. Accurately determining which patients will have a pCR will improve prognostic information for patients and families, confirm lack of response to nCRT, or avoid surgery if no residual tumor is present. Imaging, endoscopy, and liquid biomarkers have all failed to detect pCR without performing an esophagectomy.

**Methods:**

In this study, we are enrolling patients with esophageal adenocarcinoma and squamous cell carcinoma. Patients will undergo standard evaluation including CT scans, laboratory tests, endoscopy with biopsies, and evaluation by a thoracic surgeon. Tissue biopsy is required for enrollment that will be sent for BH3 profiling and metabolomics. Patients will be treated with standard nCRT followed by surgery. Patients with metastatic disease are not eligible. Surgery at the National Cancer Institute will be minimally-invasive robotic surgery. Patients will remain on study indefinitely with regular clinic visits and imaging tests.

**Discussion:**

The mitochondria are critically involved in the intrinsic pathway apoptosis. Bcl-2 homology domain 3 (BH3) profiling is a technique to measure a cell’s susceptibility to apoptosis. BH3 profiling measures the relative interactions of proteins that induce or block apoptosis. The collective balance of these proteins determines whether a cell is near the threshold to undergo apoptosis. If the cell is near this threshold, then the tumor may be more likely to die when treated with nCRT. The mitochondria secrete metabolites that may be detectable as biomarkers. Metabolomics is a global assessment of all metabolite changes that has been performed for detection, monitoring, prognosis, and treatment response in cancers. Stratification of patients based on whether pCR occurs or not may elucidate metabolomic signatures that may be associated with response. We are asking whether BH3 profiling or a metabolomic signature will correlate with tumor death after nCRT for esophageal cancer.

**Trial registration:**

NCT03223662; Clinicaltrials.gov. July 21, 2017.

**Electronic supplementary material:**

The online version of this article (10.1186/s12876-018-0823-x) contains supplementary material, which is available to authorized users.

## Background

Esophageal adenocarcinomas (EAC) and esophageal squamous cell cancers (ESCC) accounted for nearly 17,000 new diagnoses and 16,000 deaths in 2016 [1]. EAC is the dominant histology in the United States and its incidence continues to rise [2]. Neoadjuvant chemoradiotherapy (nCRT) followed by surgical removal of the esophagus (esophagectomy) demonstrated a 47% five-year survival compared to a 34% five-year survival for patients who underwent surgery alone in the CROSS trial [3]. Patients having received nCRT who had no residual tumor at the time of the esophagectomy, referred to as a pathological complete response (pCR), were most likely to achieve long-term survival [4–8]. pCR occurs in about 20% of patients with EAC and 50% of patients with ESCC [9–13]. Accurately predicting response to neoadjuvant therapy may improve prognostic information for patients and families, confirm lack of response to ineffective regimens, and avoid esophagectomy when pCR occurs [14]. Currently, the only means to identify pCR are 18F-FDG-PET scan and endoscopy with biopsies. These tests have been studied extensively and neither reliably predict pCR with a combined positive predictive value of 36% [15, 16]. To date, no reliable biomarker has been developed. Therefore, the only accurate assessment of pathological response is obtained via examination of the excised esophagus [10, 17]. We hypothesize that a metabolomic signature or bcl-2 homology domain 3 (BH3) profiling will correlate with pCR.

Metabolomics is a method of global detection of small molecule metabolites. It allows for analysis of metabolite changes under many conditions including stress, changes in diet, treatment response, or other biological conditions. Increasingly, metabolomics has been used in patients with cancer for tumor detection as well as assessment of prognosis, progression, and treatment response [18]. Clinical examples of utility of metabolomics include monitoring the progression of prostate cancer [19, 20], determining prognosis of glioblastoma and anaplastic astrocytoma [21], and predicting response to imatinib in chronic myelogenous leukemia [22]. We hypothesize that metabolic differences exist between patients who achieve a pCR versus those who do not, therefore, a metabolomic signature should be associated with differences in pathological responses.

Bcl-2 homology domain 3 (BH3) profiling is a technique to measure a cell’s readiness to die by the intrinsic pathway of apoptosis. Apoptosis is a mechanism of cell death that prevents damaged cells from becoming cancerous. The inability of damaged cells to undergo apoptosis is a well-established hallmark of cancer [23]. The intrinsic pathway of apoptosis induces mitochondrial outer membrane permeabilization (MOMP) which results in a series of events culminating in cell death. MOMP is regulated by the Bcl-2 family of proteins that are broadly divided into pro-apoptotic and anti-apoptotic proteins. The interactions of certain Bcl-2 proteins occur at the BH3 domains [24, 25]. BH3 profiling measures the relative interactions of pro- and anti-apoptotic proteins to determine whether a tumor cell is near the threshold to activate apoptosis. Cells are considered ‘primed’ or ‘unprimed’ based on whether they are near or far from this threshold, respectively [24–27]. BH3 profiling has been used to successfully predict response to chemotherapy and resistance to targeted therapy in both clinical samples and laboratory cell culture models [28]. BH3 profiling significantly correlated with progression-free survival after treatment with carboplatin and paclitaxel in patients with ovarian adenocarcinoma [28]. We hypothesize that BH3 profiling will correlate with pCR after nCRT for patients with esophageal cancers.

Approximately 50–70% of esophageal cancers harbor mutations in the TP53 gene (p53 protein) which is the most commonly mutated gene in cancer [29]. These mutations result in both loss of tumor suppressor activity and acceleration of tumor growth [30, 31]. EAC patients with p53 mutations respond poorly to chemotherapy and have worse outcomes after either surgery alone or nCRT [32, 33]. Evaluation of p53 mutations will help determine whether patients need different treatment strategies based on p53 status.

The primary aim of this trial is to determine whether a metabolomic signature and BH3 profiling correlates with pCR. The secondary aim of this trial is to determine whether p53-mutational status of the tumors alters the metabolomic signatures or BH3 profiling. Additional secondary aims are to correlate these findings with overall and disease-free survivals. In summary, we anticipate that BH3 profiles or metabolomic signatures will be associated with treatment response to nCRT in esophageal cancer to serve as a basis for precision-based, personalized strategies for future treatment.

## Methods

### Study Type

Prospective, observational, two-armed (EAC and ESCC) trial.

### Aim

#### Primary objective

- To determine whether a metabolomic signature or BH3 profiles correlates with pathological complete response (pCR) after neoadjuvant chemoradiotherapy (nCRT) for patients with esophageal adenocarcinoma (EAC) or squamous cell carcinoma (ESCC).

#### Secondary objectives

- To examine if metabolomic signatures or BH3 profiles correlate with disease-free survival (DFS) or overall survival (OS).
- To explore whether specific p53 mutations correlate with metabolomic signatures or BH3 profiles.

### Design

#### Inclusion criteria

- Histologically confirmed EAC or ESCC.
- Stage appropriate for treatment by both nCRT and surgery.
- Disease deemed resectable by surgeon assessment and imaging.

#### Exclusion criteria

- Patients for whom nCRT followed by surgery is not the appropriate management:
  - Early stage disease requiring local therapy without nCRT.
  - Metastatic disease.
- Performance status that precludes nCRT and/or surgery.
- Biopsy prior to starting nCRT not obtainable.

### Statistics

The primary objective is to determine whether a metabolomic signature in tumor, blood, or urine or whether BH3 profiling of pre-neoadjuvant tumor biopsy correlates with the outcome of pCR after nCRT for patients with EAC or ESCC. The measurement of the primary endpoint is whether or not viable tumor is present after surgical resection – pCR. Similarly, a secondary objective is to identify whether metabolomic signatures in tumor, blood, or urine or BH3 profiling in tumor of EAC and ESCC patients correlate with major responses (Mandard score of 1 and 2) versus minimal response (Mandard score 3–5). This analysis is also based on pathological findings of the surgical resection. The patients with major responses include < 10% viable tumor and pCR versus patients with any grade over 10% viable tumor. Another secondary objective is descriptive to evaluate metabolomic profiles of these patients as an exploratory analysis to determine whether certain pathways are significantly upregulated in esophageal cancer.

#### Sample size calculations

Since this trial is an exploratory biomarker trial, the true number of patients to power this study is unknown. Sreekumar and colleagues compared 16 prostate normal tissue samples to 12 samples of localized prostate cancer to 14 metastatic prostate samples and successfully identified metabolites associated with progression in prostate cancer [20].

We will plan to accrue 10 patients with pCR for both EAC and ESCC in order to have a minimal number of patients with pCRs to compare against the other subjects. For patients with EAC or ESCC, the percentage of patients with a pCR after nCRT is well-documented and significantly different. Therefore, patients with EAC and ESCC will be evaluated independently in two cohorts. Patients with EAC are reported to have 17–27% of patients pCR. Assuming 20% of patients have a pCR with EAC, 66 patients will be accrued in order to have an 86% probability of obtaining 10 patients with pCR. Thus, the accrual goal for Arm 1 for EAC will be set at 66 evaluable patients, and will have an accrual ceiling of 80 patients to allow for up to 14 inevaluable cases. Patients with ESCC are reported to have 40–64% of pCR after neoadjuvant CRT. Assuming 40% of patients have a pCR with ESCC, 32 evaluable patients will be accrued in order to have an 88% probability of obtaining 10 patients with pCR. Thus, the accrual goal for arm 2 will be set at 32 evaluable patients, and will have an accrual ceiling of 40 patients to allow for unevaluable cases. The overall accrual ceiling of the entire study will be 120 patients to allow for to 22 unevaluable patients.

To allow for a small number of unevaluable patients, the accrual ceiling will be set to 120 patients for the entire study. The accrual ceiling will be 80 patients for EAC. If one patient every month enrolls onto this study, accrual is expected to be completed in 6–7 years. The accrual ceiling will be 40 patients for ESCC. If one patient every 2 months enrolls onto this study, the accrual is expected to be completed in 6 years.

The secondary outcomes will be the association of overall survival (OS), disease-free survival (DFS), pathological stage (ypStage), and p53 mutational status with a metabolic signature. Additionally, the patients will be divided by pathological major response (Mandard 1–2) compared to minor or no response (Mandard 3–5). This analysis is similar to comparison of pCR to non-pCR, however, patients with < 10% viable tumor will be included in the favorable group; therefore, this group will be slightly larger than 10 patients with pCR. The OS and DFS will be calculated by Dr. Seth Steinberg using Kaplan-Meier and log-rank tests. The final, pathological stage will be reviewed by the PI prior to any analysis. Given that multiple stages are possible, the additional subgroup analysis of ypStage will be reported as descriptive statistics only without metabolic analysis.

#### Analysis of data

Analyses involving the actual metabolomics profiles, as well as the analyses involving metabolic signatures, will be done in conjunction with the company, Metabolon, who will receive, process, and analyze deidentified patient samples (www.metabolon.com). Metabolon uses an authenticated biochemical reference library as standards for known metabolites with LC/MS methodology. This library enables comparisons to identify differences in metabolites in our patient cohorts. The bioinformatic analysis of metabolomic profiles between those with a pCR and the other patients will be done by propriety software that compares the mass spectral ion features of their library to our cohorts. This data is further processed by mapping to known cellular pathways.

### Intervention

Assessment prior to initiation of neoadjuvant chemoradiotherapy:

- Complete history and physical examination.
- Nutritional assessment and routine laboratory evaluations.
- CT and/or PET-CT scan of chest, abdomen and pelvis.
- Esophagogastroduodenoscopy (EGD) with confirmation of histology and specimens for metabolomic and BH3 profiling.
  After neoadjuvant therapy, patients will undergo standard preoperative assessment including:
- Complete history and physical examination with clinical assessment for fitness for surgery.
- Pulmonary function tests (PFTs) if indicated.
- Cardiac evaluation and/or EKG if indicated.
- CT, PET or PET-CT scan of chest, abdomen and pelvis.
- Routine preoperative laboratory evaluations.

Surgery and post-operative care:

- Robotically-assisted, minimally-invasive esophagectomy (RAMIE) will be performed if feasible.
- For those for whom a minimally-invasive procedure is contraindicated, a traditional open approach will be performed.
- Jejunostomy tubes are placed in all patients for post-operative nutritional support.
- Patients will receive routine post-esophagectomy care including initial monitoring in ICU.

Follow-up of Study

- Routine clinic appointments and CT scans will be performed at 3, 6, 9, 12, 18, and 24 months then yearly for at least 5 years.
- Patients will be followed for the secondary endpoints of disease-free survival and overall survival.
- Patients will continue surveillance and may remain on study indefinitely.

## Discussion

Esophageal cancer is an increasing health burden both in the United States and worldwide. The phase III CROSS trial noted a survival benefit of neoadjuvant chemoradiotherapy (nCRT) followed by surgery, but the patients who experienced the best outcomes in this trial had almost no residual tumor after nCRT [3]. Evaluation after nCRT by PET scans and endoscopy have been studied by multiple groups which have consistently reported that these tests correlate poorly with final pathology [13, 15, 16, 34]. Similarly, biomarker discovery has not yielded a predictive marker for treatment response. Currently, the only method to assess treatment response after nCRT is removal of the esophagus [10, 17]. nCRT and surgery are standard recommendations for treatment of locally-advanced esophageal cancers, however, this treatment strategy is associated with significant morbidity, especially in older patients who often have additional comorbidities. The ability to predict whether nCRT is efficacious has several advantages that could significantly improve patient outcomes by personalizing treatment strategies. First, predicting which patients will respond will help counsel patients and families about expected outcomes. Second, determining if nCRT is not effective will allow discontinuation of toxic regimens in order to proceed to surgery more quickly. Third, if patients achieve pathological complete response (pCR), then surgery is not indicated and these patients can avoid removal of the esophagus.

The most common mechanism of cell death secondary to chemotherapy and radiation therapy is activation of the intrinsic pathway of apoptosis. Apoptosis is a programmed cell death that kills the cell in an orderly manner without induction of inflammation. The intrinsic pathway activates pre-formed proteins that alter the mitochondria leading to cell death. In an individual patient or an individual tumor, the mitochondria may be susceptible or resistant to apoptosis. This concept is analogous to a cell or mitochondria being ‘primed’ or ‘unprimed’ for cell death [23–27]. The mitochondria generate energy for cellular function and building blocks for tumor growth. These processes are associated with several metabolic pathways that secrete metabolites with each step. Differences in these pathways may impart the resistance to apoptosis and these differences should secrete different levels of metabolites that could be detected by metabolomics. The metabolic signature of a tumor sensitive to nCRT should be quite different than a resistant tumor [18, 22]. Unlike the global detection of metabolite secretion by metabolomic analysis, BH3 profiling directly measures the functional interactions of the proteins that both induce and block apoptosis. For example, if a tumor contains a high amount of functional Bcl-xL, an anti-apoptotic protein, this tumor may be resistant to nCRT and BH3 profiling should correlate with lack of efficacy of nCRT [27]. Additionally, BH3 profiling provides a test that can be performed within 24 h of biopsy which makes this assessment ideal for patient treatment decisions. Despite the differences between metabolomic signatures and BH3 profiling, they are expected to provide independent evaluations of mitochondrial susceptibility to cell death.

Predicting apoptosis by metabolomic signatures or BH3 profiling focuses on downstream cellular processes independent of tumor mutational status. However, esophageal cancers, similar to other cancers induced by environmental exposures, have a high burden of somatic mutations [35]. Mutations in the TP53 gene are detected in greater than 50% of esophageal cancers whereas the next most common mutations occur in less than 12% of esophageal cancers [32]. Wild-type p53 has multiple functions including the induction of apoptosis whereas p53 mutations may block apoptosis and therefore ‘unprime’ a tumor cell. p53 mutations may affect both metabolomic signatures as well as BH3 profiling [30, 31]. Therefore, all tumors will be sequenced for TP53 gene mutations and data analysis will account for the mutation type.

Our goal is to determine whether metabolomic signatures or BH3 profiling correlate with treatment response to nCRT. If these techniques can achieve this goal, we may improve patient care by personalizing treatment regimens. Furthermore, if these techniques are successful, future trials will be based on altering the mitochondrial threshold for apoptosis to increase the susceptibility for standard therapeutics. Mitochondrial priming is dynamic, therefore, its threshold for apoptosis can be decreased by selecting tumor specific therapies. For example, blocking Bcl-xL in a tumor that is reliant on this anti-apoptotic protein may ‘prime’ that cell for death. Alternatively, if p53 mutational status blocks apoptosis, inhibition of p53 may be required to increase the efficacy of standard nCRT. This current observational trial will help design future interventional trials aimed at increasing the pathological response rates which will improve the overall survival of patients with esophageal cancers.

### Trial status

Open.

## Additional file

Additional file 1: Consent to Participate in a Clinical Research Study: ‘Metabolomic and BH3 Profiling of Esophageal Cancers: Identification of Novel Assessment Methods of Treatment Response for Precision Therapy’. Institutional Review Board-approved National Cancer Institute patient consent to participation in this study. (DOC 122 kb)
