# Supplementary material for: Metabolomic and BH3 profiling of esophageal cancers: novel assessment methods for precision therapy
Source: BMC Gastroenterol. 2018 Jun 22;18:94. doi: 10.1186/s12876-018-0823-x (PMC6013848; doi:10.1186/s12876-018-0823-x)
Supplement: Supplementary file 1 — Consent to Participate in a Clinical Research Study: ‘Metabolomic and BH3 Profiling of Esophageal Cancers: Identification of Novel Assessment Methods of Treatment Response for Precision Therapy’. Institutional Review Board-approved National Cancer Institute patient consent to participation in this study. (DOC 122 kb) [file 12876_2018_823_MOESM1_ESM.doc]

# INTRODUCTION

We invite you to take part in a research study at the National Institutes of Health (NIH).

First, we want you to know that:

Taking part in NIH research is entirely voluntary**.**

You may choose not to take part, or you may withdraw from the study at any time. In either case, you will not lose any benefits to which you are otherwise entitled. However, to receive care at the NIH, you must be taking part in a study or be under evaluation for study participation.

You may receive no benefit from taking part. The research may give us knowledge that may help people in the future.

Second, some people have personal, religious or ethical beliefs that may limit the kinds of medical or research treatments they would want to receive (such as blood transfusions). If you have such beliefs, please discuss them with your NIH doctors or research team before you agree to the study.

Now we will describe this research study. Before you decide to take part, please take as much time as you need to ask any questions and discuss this study with anyone at NIH, or with family, friends or your personal physician or other health professional.

# Why is this study being done?

We would like to determine if there are markers in your tumor, blood, or urine that are related to the outcome of the neoadjuvant chemoradiotherapy you will receive. Neoadjuvant therapy is treatment given to shrink a tumor before surgery.

# Why are you being asked to take part in this study?

You are being asked to participate in this study because you have an esophageal adenocarcinoma or squamous cell carcinoma.

# How many people will take part in this study?

Up to 120 individuals will be asked to participate in this study.

# Description of Research Study

## Before you begin the study

Before you enroll in the study you will need to have the following exams and tests to make sure you are eligible for this study: standard lab tests, imaging studies, and a physical exam to be sure that it would be safe for you to proceed with chemoradiotherapy followed by surgery. You will be asked to provide a sample of your tumor from a previous surgery so that we confirm your diagnosis. If sample is not available, you will need to have a biopsy of your tumor. If you are a woman who can have children, pregnancy test will be done, because pregnant women cannot participate in the study.

## During the study

First, you will undergo a baseline evaluation (if these tests were already performed at screening, we probably will not need to repeat them):

- History, physical examination and assessment of your activity level
- EGD (Esophagogastroduodenoscopy - is a procedure to examine the esophagus, stomach and first portion of the small intestine: lighted tube will be inserted into your mouth and go down to the small intestine). Biopsies of tumor and normal tissue will be taken for research studies
  - The biopsy by endoscopy by our team is the critical step to obtain tissue. This biopsy is necessary for the study, as it is important for deciding the best operation for you. After this endoscopy, you will usually return to your referring doctors for standard chemotherapy and radiation. After that treatment, you will return to the NIH for esophagectomy. Given that you will often be treated by us at the NIH and your home doctor, we do ask that you enroll on another study that allows us to treat you.
- PET/CT scan
- Routine blood tests
- Pregnancy test
- Nutritional assessment
- Research blood and urine tests
- PFT (pulmonary test) if medically indicated
- EUS (Endoscopic ultrasound) if medically indicated.

After that you will have neoadjuvant chemoradiotherapy here at NIH Clinical Center or with your local physician.

After chemoradiotherapy you must return to the NIH Clinical Center for surgery and recovery. During surgery, we will take tumor and normal tissue biopsies for research studies. You will have blood and urine collected for research on Day 7 after surgery.

You will undergo a robotically-assisted, minimally-invasive esophagectomy (RAMIE) if feasible at the NCI in which the operation is performed with cameras and scopes. For those whom a scope procedure is contraindicated or for institutions that do not perform these procedures, a traditional open approach will be performed with incisions in the abdomen, chest, and neck.

The surgical approach depends on tumor location. The approach will be different for adenocarcinoma versus squamous cell carcinoma. Most adenocarcinoma are located in the lower esophagus near the stomach which requires an abdominal incision with either a chest or neck incision. In contrast, most squamous cell carcinomas are in the middle of the esophagus and require an incision in the chest followed by an incision in both the abdomen and neck. With both operations, most of the esophagus and top of the stomach are removed. The remainder of the stomach is pulled into the chest and connected to the remaining esophagus.

Feeding tubes are placed in all patients at the NCI. Feeding by the tube will be started a few days after the operation until you are eating enough to maintain enough calories which typically takes 10-14 days. Feeding tubes are continued after discharge for a variable length of time depending on your nutritional needs.

Following the surgery, we will invite you every 3 month during the first year, every 6 months during the second year and yearly after that for post-operative evaluation and research tests:

- PET/CT scan
- Routine blood tests
- Nutritional assessment
- Research blood and urine tests

# Birth Control

If you are a woman who is breast feeding or pregnant, you may not take part in the study. It is best to avoid radiation and chemotherapy exposure to unborn children. If you are a woman who can become pregnant, or are the partner of a woman who can become pregnant, you will need to practice an effective form of birth control before starting study treatment and during study treatment. If you think that you or your partner is pregnant, you should tell your study doctor or nurse at once.

Effective forms of birth control include:

- abstinence
- intrauterine device (IUD)
- hormonal [birth control pills, injections, or implants]
- tubal ligation
- vasectomy

# Risks or Discomforts of Participation

If you choose to take part in this study, there is a risk that:

- You may lose time at work or home and spend more time in the hospital or doctor’s office than usual
- You may be asked sensitive or private questions which you normally do not discuss

## Neoadjuvant therapy and Surgery

The side effects from the neoadjuvant therapy and surgery are the same as you would have if you received standard therapy for an esophageal adenocarcinoma or squamous cell carcinoma at any medical center.

## Research Procedure Risks

### Blood Draw

Side effects of blood draws include pain and bruising in the area where the needle was placed, lightheadedness, and rarely, fainting. When large amounts of blood are collected, low red blood cell count (anemia) can develop.

### Urine collection

There are no physical risks or discomforts associated with urine collection.

### EGD and biopsy

The side effects from the EGD and biopsy may include discomfort in your throat, bleeding, perforation and infection.

### Surgical Procedure

An esophagectomy carries significant post-surgical complications. The main risks that are life threatening are heart attacks and blood clots. Both conditions are rare. The risk of heart attack is evaluated by assessing your heart before the operation with standard tests. The risk of blood clots is reduced with use of blood thinners and by walking after the operation. The next most serious complications are pneumonia and leak of the connection between the esophagus and the stomach. Pneumonia is an infection of the lungs that is treated with antibiotics. It ranges from serious to minor but will be monitored closely. Occasionally, the breathing machine is necessary to recover from pneumonia. A leak requires intervention to fix. Usually, we will perform an endoscopy and place a stent to let the leak heal. Occasionally, we have to operate to fix it. Loss of the stomach connected to the esophagus is very rare, but if it occurs, re-operation is necessary.

Multiple additional complication can occur but usually are managed without significant problems. Infections in the incisions are drained or treated with antibiotics. Bladder or urine infections are treated with antibiotics. Abnormal heart rhythms are common and usually managed with medications. Blood clots in the legs are usually managed with stronger blood thinners.

### Privacy Risks related to the release of genetic information:

- Your privacy is very important to us and we will use many safety measures to protect your privacy. However, despite all the safety measures that we will use, we cannot guarantee that your identity will never become known. Although your genetic information is unique to you, you do share some genetic information with your children, parents, brothers, sisters, and other blood relatives. Consequently, it may be possible that genetic information from them could be used to help identify you. Similarly, it may be possible that genetic information from you could be used to help identify them.
- While the controlled-access databases developed for this project will not contain information that is traditionally used to identify you, such as your name, address, telephone number, or social security number, people may develop ways in the future that would allow someone to link your genetic or medical information in our databases back to you. For example, someone could compare information in our databases with information from you (or a blood relative) in another database and be able to identify you (or your blood relative). It also is possible that there could be violations to the security of the computer systems used to store the codes linking your genetic and medical information to you.
- Since some genetic variations can help to predict the future health problems of you and your relatives, this information might be of interest to health providers, life insurance companies, and others. Patterns of genetic variation also can be used by law enforcement agencies to identify a person or his/her blood relatives. Therefore, your genetic information potentially could be used in ways that could cause you or your family distress, such as by revealing that you (or a blood relative) carry a genetic disease.
- There also may be other privacy risks that we have not foreseen.

There are state and federal laws that protect against genetic discrimination. There is also a federal law called the Genetic Information Nondiscrimination Act (GINA). In general, this law makes it illegal for health insurance companies, group health plans, and most employers to discriminate against you based on your genetic information. However, it does not protect you against discrimination by companies that sell life insurance, disability insurance, or long-term care insurance. GINA also does not apply to members of the United States military, to veterans obtaining health care through the Veteran’s Administration or the Indian Health Service. Lastly, GINA does not forbid insurance medical underwriting based on your current health status.

# Potential Benefits of Participation

## Are there benefits to taking part in this study?

You will receive appropriate medical and surgical therapy, but we do not expect you to gain personal benefit from enrollment in this study.

## Will my genetic information be shared with me?

The genetic analyses performed in various laboratories under this protocol are for research purposes only and they are not as sensitive as the tests that are performed in a laboratory that is certified to perform genetic testing for clinical purposes (CLIA certification). Observed changes may or may not be valid. Therefore, we do not plan to inform you of the results of testing on the tissue and blood that is performed in our research lab. However, in the unlikely event that clinically relevant incidental findings are discovered, you will be contacted.

In this case, we will ask you to provide additional blood sample to be sent to a CLIA certified laboratory. If the research findings are verified in the CLIA certified lab, you will be referred to an NCI CCR Genetics Branch certified genetic health care provider for the disclosure of the results. This is the only time during the study when incidental findings will be returned to you.

# Alternative Approaches or Treatments

Instead of being in this study, you have these options:

- Getting treatment or care for your esophageal cancer at a different facility without being in a study

# Taking part in another study that you are eligible for.

# Getting comfort care, also called palliative care. This type of care helps reduce pain, tiredness, appetite problems and other problems caused by the cancer. It does not treat the cancer directly. Instead, it tries to improve how you feel. Comfort care tries to keep you as active and comfortable as possible.

# Please talk to your doctor about these and other options.

# Research Subject’s Rights

## What are the costs of taking part in this study?

If you choose to take part in the study, the following will apply, in keeping with the NIH policy:

- You will receive study treatment at no charge to you. This may include surgery, medicines, laboratory testing, x-rays or scans done at the Clinical Center, National Institutes of Health (NIH), or arranged for you by the research team to be done outside the Clinical Center, NIH if the study related treatment is not available at the NIH.
- There are limited funds available to cover the cost of some tests and procedures performed outside the Clinical Center, NIH. You may have to pay for these costs if they are not covered by your insurance company.
- Medicines that are not part of the study treatment will not be provided or paid for by the Clinical Center, NIH.
- Once you have completed taking part in the study, medical care will no longer be provided by the Clinical Center, NIH.

## Will your medical information be kept private?

We will do our best to make sure that the personal information in your medical record will be kept private. However, we cannot guarantee total privacy. Organizations that may look at and/or copy your medical records for research, quality assurance, and data analysis include:

- The National Cancer Institute (NCI) and other government agencies, like the Food and Drug Administration (FDA), which are involved in keeping research safe for people.
- National Cancer Institute Institutional Review Board

A description of this clinical trial will be available on http://www.Clinicaltrials.gov, as required by U.S. Law. This Web site will not include information that can identify you. At most the Web site will include a summary of the results. You can search this Web site at any time.

# Stopping Therapy

Your doctor may decide to stop your therapy for the following reasons:

- if he/she believes that it is in your best interest
- If surgery is no longer appropriate because your cancer spread to other parts of the body or you are not able to safely undergo an operation.
- if new information shows that another treatment would be better for you
- if you become pregnant

In this case, you will be informed of the reason therapy is being stopped.

You can stop taking part in the study at any time. However, if you decide to stop taking part in the study, we would like you to talk to the study doctor and your regular doctor first.

If you decide at any time to withdraw your consent to participate in the trial, we will not collect any additional medical information about you. If you withdraw your consent and leave the trial, any samples of yours that have been obtained for the study and stored at the NCI can be destroyed upon request. However, any samples and data generated from the samples that have already been distributed to other researchers or placed in the research databases cannot be recalled and destroyed.

# Certificate of Confidentiality

To help us protect your privacy, we have obtained a Certificate of Confidentiality from the National Institutes of Health. The researchers can use this Certificate to legally refuse to disclose information that may identify you in any federal, state, or local civil, criminal, administrative, legislative, or other proceedings, for example, if there is a court subpoena. The researchers will use the Certificate to resist any demands for information that would identify you, except as explained below.

The Certificate cannot be used to resist a demand for information from personnel of the United States Government that is used for auditing or evaluation of Federally-funded projects or for information that must be disclosed in order to meet the requirements of the federal Food and Drug Administration (FDA).

You should understand that a Certificate of Confidentiality does not prevent you or a member of your family from voluntarily releasing information about yourself or your involvement in this research. If an insurer, employer, or other person obtains your written consent to receive research information, then the researchers may not use the Certificate to withhold that information.

The Certificate of Confidentiality will not protect against the required reporting by hospital staff of information on suspected child abuse, reportable communicable diseases, and/or possible threat of harm to self or others.

# Use of Data for Future Research

To advance science, it is helpful for researchers to share information they get from studying human samples. They do this by putting it into one or more scientific databases, where it is stored along with information from other studies. A researcher who wants to study the information must apply to the database and be approved. Researchers use data stored in scientific databases to advance science and learn about health and disease.

We plan to keep some of your data that we collect and use them for future research and share them with other researchers. We will not contact you to ask about each of these future uses. These data will be stripped of identifiers such as name, address or account number, so that they may be used for future research on any topic and shared broadly for research purposes. Your data will be used for research purposes only and will not benefit you. It is also possible that the stored data may never be used. Results of research done on your data will not be available to you or your doctor. It might help people who have cancer and other diseases in the future.

If you do not want your stored data used for future research, please contact us in writing and let us know that you do not want us to use your data. Then your data will not be used for future research. However, it may not be possible to withdraw or delete data once they have been shared with other researchers.

**OTHER PERTINENT INFORMATION**

**1. Confidentiality.** When results of an NIH research study are reported in medical journals or at scientific meetings, the people who take part are not named and identified. In most cases, the NIH will not release any information about your research involvement without your written permission. However, if you sign a release of information form, for example, for an insurance company, the NIH will give the insurance company information from your medical record. This information might affect (either favorably or unfavorably) the willingness of the insurance company to sell you insurance.

The Federal Privacy Act protects the confidentiality of your NIH medical records. However, you should know that the Act allows release of some information from your medical record without your permission, for example, if it is required by the Food and Drug Administration (FDA), members of Congress, law enforcement officials, or authorized hospital accreditation organizations.

**2. Policy Regarding Research-Related Injuries.** The Clinical Center will provide short-term medical care for any injury resulting from your participation in research here. In general, no long-term medical care or financial compensation for research-related injuries will be provided by the National Institutes of Health, the Clinical Center, or the Federal Government. However, you have the right to pursue legal remedy if you believe that your injury justifies such action.

**3. Payments.** The amount of payment to research volunteers is guided by the National Institutes of Health policies. In general, patients are not paid for taking part in research studies at the National Institutes of Health. Reimbursement of travel and subsistence will be offered consistent with NIH guidelines.

**4. Problems or Questions.** If you have any problems or questions about this study, or about your rights as a research participant, or about any research-related injury, contact the Principal Investigator, Taylor Ripley, M.D., Building 10, Room 4-3952, Telephone: 240-760-6237. You may also call the Clinical Center Patient Representative at 301-496-2626. If you have any questions about the use of your specimens or data for future research studies, you may also contact the Office of the Clinical Director, Telephone: 240-760-6070.

**5. Consent Document.** Please keep a copy of this document in case you want to read it again.

| **COMPLETE APPROPRIATE ITEM(S) BELOW:** | | | | |
| --- | --- | --- | --- | --- |
| **A. Adult Patient’s Consent** | | **B. Parent’s Permission for Minor Patient.** | | |
| I have read the explanation about this study and have been given the opportunity to discuss it and to ask questions. I hereby consent to take part in this study. | | I have read the explanation about this study and have been given the opportunity to discuss it and to ask questions. I hereby give permission for my child to take part in this study.  (Attach NIH 2514-2, Minor’s Assent, if applicable.) | | |
|  |  |  | |  |
| Signature of Adult Patient/  Legal Representative | Date | Signature of Parent(s)/ Guardian | | Date |
| Print Name | | Print Name | | |
| **C. Child’s Verbal Assent (If Applicable)**  The information in the above consent was described to my child and my child agrees to participate in the study. | | | | |
|  |  | |  | |
| Signature of Parent(s)/Guardian | Date | | Print Name | |
| **THIS CONSENT DOCUMENT HAS BEEN APPROVED FOR USE  FROM JULY 12, 2017 THROUGH JULY 11, 2018.** | | | | |
|  |  | |  |  |
| Signature of Investigator | Date | | Signature of Witness | Date |
| Print Name | | | Print Name | |
